# A Misrepresentation Corrected

**Published:** 1888-08

**Authors:** 


### A Misrepresentation Corrected.

Soon after the meeting of the International Medical Congress, in Washington, in September, 1887, the statement was made by a Washington paper, and subsequently copied into several other papers, and was even repeated from the pulpit, to the effect that $6,000 of the appropriation of $10,000 made by Congress to aid in defraying the necessary expenses of the meeting, were expended by the Local Committee of Arrangements for wines and liquors. As the terms of the congressional appropriation required the money to be expended under the direction of the Secretary of the Treasury, that high officer of the government has recently transmitted to the Speaker of the House of Representatives, a full itemized bill for the amount. If those who have been so ready to accuse the medical men of squandering the public money for strong drink, will take the trouble to examine this bill they will see that not one dollar of the money drawn from the National Treasury was spent for drinks or refreshment of any kind.

